# Empagliflozin Leads to Faster Improvement in Arterial Stiffness Compared to Dapagliflozin: A Double-Blind Clinical

**DOI:** 10.3390/life15050802

**Published:** 2025-05-18

**Authors:** Erick González Campos, Fernando Grover Páez, Carlos Gerardo Ramos Becerra, Luis Ricardo Balleza Alejandri, Daniel Osmar Suárez Rico, Ernesto Germán Cardona Muñoz, Sara Pascoe González, María Guadalupe Ramos Zavala, Alberto Beltrán Ramírez, Jesús Jonathan García Galindo, David Cardona Müller

**Affiliations:** 1 Department of Physiology, University Health Sciences Center, Universidad de Guadalajara, Guadalajara 44340, Mexico; erick.gonzalez7206@alumnos.udg.mx (E.G.C.); carlos.rbecerra@academicos.udg.mx (C.G.R.B.); luis.balleza3286@alumnos.udg.mx (L.R.B.A.); daniel.suarez@academicos.udg.mx (D.O.S.R.); german.cardona@academicos.udg.mx (E.G.C.M.); sara.pascoe@academicos.udg.mx (S.P.G.); alberto.beltran@academicos.udg.mx (A.B.R.); jonathan.garcia@academicos.udg.mx (J.J.G.G.); david.cardona@academicos.udg.mx (D.C.M.); 2 Arterial Stiffness Laboratory, Experimental and Clinical Therapeutics Institute, Department of Physiology, University Health Sciences Center, Universidad de Guadalajara, Guadalajara 44340, Mexico

**Keywords:** cf-pulse wave velocity, arterial stiffness, type 2 diabetes, SGLT2i

## Abstract

(1) Background: Arterial stiffness, often measured by carotid–femoral pulse wave velocity (cf-PWV), is crucial in cardiovascular disease. Dapagliflozin has shown rapid effects on arterial stiffness, but there is limited evidence of empagliflozin’s acute effects, especially in type 2 diabetes (T2D) patients. This study evaluated the acute effects of empagliflozin and dapagliflozin on arterial stiffness and blood pressure (BP). (2) Methods: A one-week double-blind randomized trial involved 30 T2D patients on stable metformin therapy. Participants received empagliflozin (25 mg/day), dapagliflozin (10 mg/day), or a placebo. Arterial stiffness was assessed via cf-PWV, and BP was measured with an automated sphygmomanometer. (3) Results: Both SGLT2 inhibitors significantly reduced cf-PWV compared to the placebo after one week (*p* < 0.05), with dapagliflozin showing a more pronounced effect. No significant differences were observed in BP changes. (4) Conclusion: Short-term treatment with SGLT2 inhibitors acutely reduces arterial stiffness in T2D patients, with empagliflozin demonstrating a stronger effect, supporting the potential vascular benefits of SGLT2 inhibitors beyond glucose control.

## 1. Introduction

In recent years, research has emphasized the role that arterial stiffness plays in the development of cardiovascular disease; its clinical assessment via pulse wave velocity is increasingly used in the examination of patients [1,2].

There is evidence indicating that arterial stiffness and, therefore, cardiovascular disease can be improved after only 48 h of treatment with dapagliflozin [3]; while there are certain drugs like β-blockers [4,5], statins [6], and inhibitors of the renin–angiotensin system [7] that also ameliorate arterial stiffness over the time, none have shown faster effects than dapagliflozin [8,9,10,11]; on the other hand, the effect of empagliflozin on arterial stiffness has been studied only in a subacute term in experimental studies or in patients with T1D [11,12]; other than in the aforementioned studies, there is no information that allows us to compare the acute effect on cf-PWV of the administration of the different SGLT2-inhibitors to patients with T2D. Therefore, this study aims to evaluate whether effects on arterial stiffness and BP are achieved acutely and whether there is a pharmacological class effect.

## 2. Materials and Methods

### 2.1. Study Design

**Figure 1:**
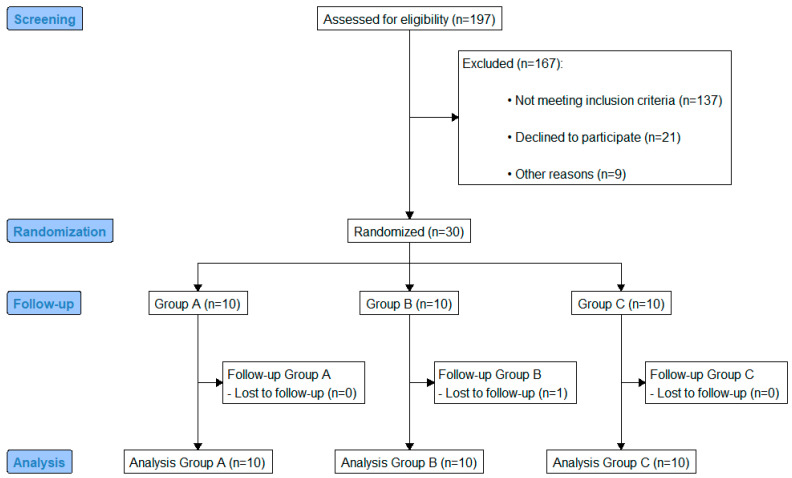
CONSORT flow diagram.

A one-week double-blind randomized clinical study that included thirty patients with T2D (15/15 male/female) was performed. A study flow diagram presenting the enrollment and randomization data is shown in Figure 1. All patients with a prior and stable metformin prescription (any standard dose for at least three months) were eligible for enrollment.

### 2.2. Randomization

The study was conducted as a double-blind trial, in which both the participants and the investigators were blinded to group allocation. Blinding was achieved through the use of sealed, opaque envelopes containing a letter-coded allocation system. The codes were maintained by an independent team not involved in the trial’s implementation or analysis, ensuring that blinding was preserved throughout the study’s duration.

Randomization was executed upon each participant’s arrival: the patient would draw a sealed opaque envelope from a box, thereby maintaining unpredictability and allocation concealment throughout the recruitment process. Participants were randomly assigned to one of three treatment groups: (1) the empagliflozin group (receiving empagliflozin 25 mg daily), (2) the dapagliflozin group (receiving 10 mg daily), and (3) the placebo group (receiving 500 mg magnesium oxide daily). All subjects voluntarily participated in this study; signed written consent to participate was requested because of the nature of the study. The protocol was approved and registered by the Research Ethics Committee of the University Center for Health Sciences, Universidad de Guadalajara (CEI/501/2020, March 2020).

### 2.3. Subjects

Inclusion criteria: (a) age between 40 and 65 years, (b) BMI < 40 kg/m^2^, (c) HbA1c between 7 and 11%, (d) under stable treatment with metformin at any standard dose (at least 3 months), (e) without a hypertension diagnosis, and (f) with stable hypertension (<140/90 mmHg) and treatment for at least 3 months. Exclusion criteria: (a) pregnant women or women without regular contraception, (b) individuals with moderate to severe renal function impairment (serum creatinine concentration > 1.5 mg/dL or GFR < 60 mL/min), (c) the presence of a clinically relevant cardiovascular, pulmonary, hematologic, or hepatic disease, or an uncontrolled thyroid disease, and/or a cancer diagnosis, (d) an active UTI, (e) treatment with insulin/sulfonylureas/TZD or beta-blockers, and (f) a recent adjustment/change (3 months) in prescription of ARAII, ACEI, diuretics, statins, nitric oxide donors, and/or digitalis.

### 2.4. Sample Size Determination

A sample size of 10 patients per group was adequate to demonstrate the difference in cf-PWV based on 1.6 m/s standard deviation (SD) of cf-PWV (Solini et al. 2017) [3], calculated with the difference of means formula for clinical trials [9], with a power of 0.80 and alpha 0.05, and considering 20% losses.

### 2.5. Endpoints

The aim of this study was to explore the effect of SGLT2i on arterial stiffness and blood pressure and the possible improvement after one week of treatment, in order to establish whether there were differences in efficacy. The co-primary endpoints were changes in cf-PWV and BP; secondary endpoints included changes in metabolic parameters.

### 2.6. Clinical Determinations

A complete history of each patient was taken, and a full medical examination was performed. Patients were advised to keep to their regular diet and usual doses of medication during the study and were subject to 8 h fasting prior to visits in which measurements were performed; abstinence from alcohol and caffeine was also required, and a daily medication checklist was provided. Subjects were randomly assigned to one of three groups (empagliflozin, dapagliflozin, and placebo). In both visits, venous blood samples were obtained, centrifuged at 2500 rpm (Beckman Coulter, Allegra X-22R, Brea, CA, USA) for 3 min, separated into serum and plasma, stored at −80 °C, and analyzed using the Erba XL-100 automated clinical chemistry analysis equipment. Glucose was assessed using the BioSystems Glucose Oxidase/Peroxidase kit, and glycated hemoglobin (HbA1c) was assessed using the BioSystems HbA1c-DIR Direct Glycated Hemoglobin kit. Arterial function assessment was performed in equal conditions in a quiet and temperature-controlled environment. Blood pressure was measured with an automated sphygmomanometer (OMRON HEM-907XL, OMRON HEALTHCARE Co., Ltd., Kyoto, Japan), and arterial stiffness evaluation was performed via carotid–femoral pulse wave velocity (cf-PWV) with DiaTecne’s PulsePen tonometer (model WPP001-ET, DiaTecne s.r.l., Milan, Italy) and PulsePen software (version WPP001-ETT-2.3.1 for Windows).

All vascular tests were performed by the same trained physician and supervised by an expert in this field.

### 2.7. Arterial Tonometry

Arterial tonometry was performed according to international recommendations (Pulse Pen, DiaTecne, Milan, Italy) [13]. Central BP was derived from the radial pressure waveform by means of a validated transfer function and averaged over three measurements. With the patient supine, ECG electrodes were placed in the right subclavian region and in the left subcostal region, and an ECG was taken; then, the tonometer was placed in the carotid region taking a signal from the carotid pulse wave; once a good quality signal had been captured, the same action was performed on the femoral artery. The last 10 cardiac complexes were saved and automatically analyzed with the software. The operator also used a tape measure to obtain the distance (in millimeters) between the carotid and femoral arteries where the pulse was taken; readings were corrected using the following formula [14]:

X_subtracted_ = 1.04X_direct_ − 0.11 × height − 0.02 (m)

All maneuvers were performed three times, and the mean was taken.

### 2.8. Statistical Analysis

Statistical analysis was performed using R Project for Statistical Computing, version 4.4.1 (https://community.chocolatey.org/packages/r.project/4.4.1 (accessed on 8 August 2024)). The Shapiro–Wilk test was applied to analyze the distribution of the data. Data obtained were given as medians and ranges for quantitative variables, and frequencies and percentages when discrete. Differences between groups at baseline were analyzed using the non-parametric Mann–Whitney U test, and the Wilcoxon rank test for intra- and intergroup analysis. A Dunn post hoc analysis was performed to compare the significance between groups.

## 3. Results

The baseline epidemiological, clinical, and hemodynamic characteristics of the participants are summarized in Table 1. No significant differences were observed between groups in terms of age, sex distribution, anthropometric parameters (height, weight, and BMI), blood pressure measurements (SBP, DBP, and MAP), HbA1c levels, triglycerides, and pulse wave velocity (cf-PWV) (all *p* > 0.05).

However, baseline glucose levels were significantly higher in the empagliflozin group compared to the dapagliflozin and placebo groups (202.37 [182.00, 304.70] mg/dL vs. 162.01 [145.00, 180.88] mg/dL and 160.98 [136.30, 180.80] mg/dL, respectively; *p* = 0.018).

At the end of the 7-day intervention, we found that patients who received the active ingredients (empagliflozin and dapagliflozin) showed a significant decrease in cf-PWV (*p* = 0.006 and *p* = 0.009, respectively), and significant changes were observed in other clinical variables including weight and BMI (*p* = 0.006 and *p* = 0.005 for empagliflozin; *p* = 0.019 and *p* = 0.022 for dapagliflozin, respectively), while in the group of patients who received a placebo, a significant increase in weight was observed (*p* = 0.019). In addition, a significant reduction was found in systolic (*p* = 0.005), diastolic (*p* = 0.008, *p* = 0.005), and mean blood pressure (*p* = 0.006) for empagliflozin and dapagliflozin, as well as in triglyceride levels in the empagliflozin group (*p* = 0.006).

Subsequently, when we compared the final net values with the Kruskal–Wallis U test, the only significant difference between the three groups was in triglyceride levels, with the empagliflozin group showing the greatest reduction. Glucose showed a tendency to decrease, although this was not statistically significant (*p* = 0.058). The rest of the variables did not show statistically significant differences (Table 2).

**Figure 2:**
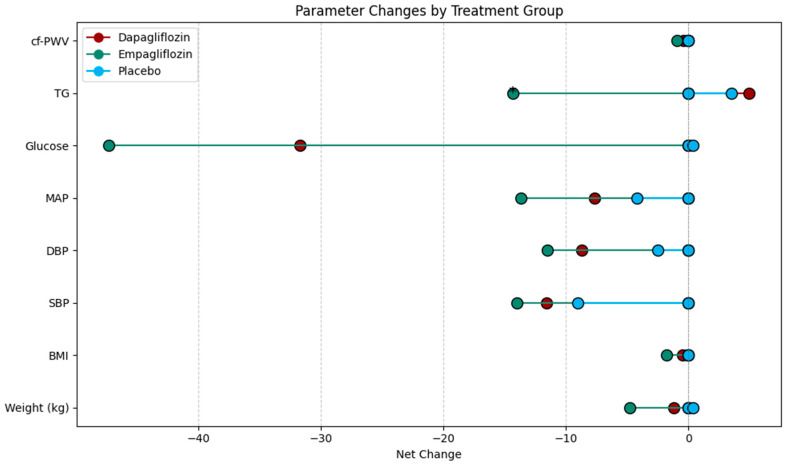
Lollipop plot showing the net change in hemodynamic and metabolic characteristics by intervention group. BMI: body mass index. SBP: systolic blood pressure. DBP: diastolic blood pressure. MAP: mean arterial pressure. TG: triglycerides. cf-PWV: carotid femoral pulse wave velocity.

In Figure 2 the net changes in the hemodynamic and metabolic characteristics of the intervention groups can be observed.

**Figure 3:**
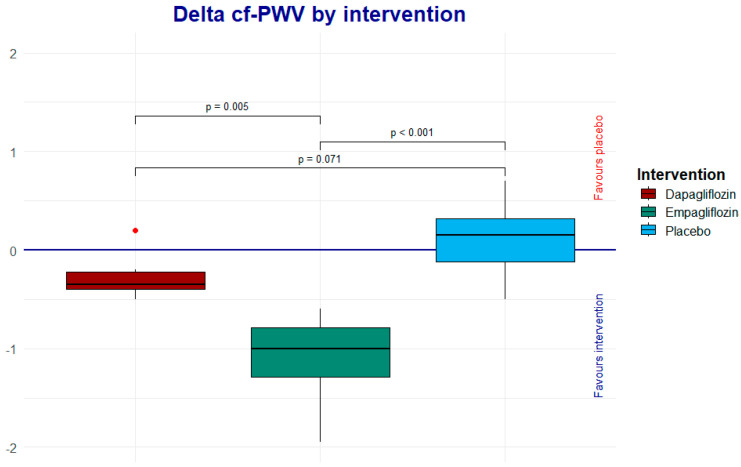
The Dunn post hoc analysis shows the net effect of each one of the interventions; the result favors empagliflozin.

The Wilcoxon rank analysis was applied to assess the intragroup change, showing a trend toward a reduction in cf-PWV in the empagliflozin group. Therefore, we performed a Dunn post hoc analysis to determine whether or not there were differences when comparing the deltas between the intervention groups. As shown in Table 3 and Figure 3, looking at the net change (Δ) in cf-PWV, empagliflozin demonstrated a greater reduction compared to dapagliflozin and the placebo (*p* < 0.001 and 0.005, respectively). The dapagliflozin group did not show a significant reduction in cf-PWV when compared to the placebo (*p* = 0.071) (Table 3, Figure 3).

**Figure 4:**
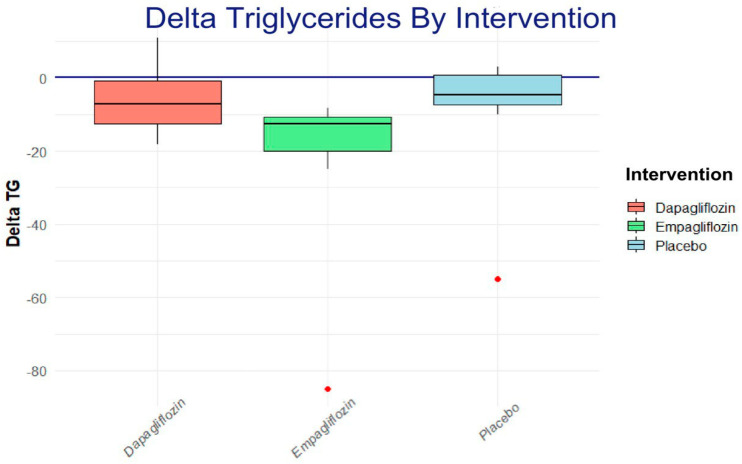
The Dunn post hoc analysis shows the net effect of each one of the interventions; the result favors empagliflozin.

To assess the intragroup change in triglycerides, a Wilcoxon rank analysis and a Dunn post hoc analysis were applied, showing a trend toward a reduction in the empagliflozin group, as shown in Figure 4.

## 4. Discussion

Sodium-glucose cotransporter 2 inhibitors (SGLT2i) have been studied for their effects on arterial stiffness, as measured by carotid–femoral pulse wave velocity (cf-PWV) [3,6,15,16]

Unlike dapagliflozin, empagliflozin shows a significant impact on arterial stiffness (cf-PWV). 

The results obtained in our study highlight a clear differential effect when comparing placebo, dapagliflozin and empagliflozin, with a statistically significant reduction in cf-PWV (especially in the latter). This effect may be related to specific mechanisms such as improvement in endothelial function, reduction in inflammation, and decreased oxidative stress, which are known effects of sodium-glucose cotransporter 2 inhibitors (SGLT2i) [3,17,18].

Regarding arterial stiffness, a meta-analysis has shown that SGLT2i may slightly reduce PWV in patients with type 2 diabetes (T2DM), although no significant decrease was observed in patients with established cardiovascular disease or cardiovascular risk factors [19]. Another study also suggests that these inhibitors improve endothelial function and arterial stiffness in diabetic individuals, although the evidence is of low certainty [20]. However, the evidence of their impact on subclinical atherosclerosis and endothelial function is heterogeneous and inconclusive [21].

No statistically significant differences were detected between dapagliflozin and placebo. Although this might suggest that dapagliflozin has no clear effect on arterial stiffness, previous studies have reported that this drug does indeed exert an effect on the arterial stiffness [3,22]; based on the above, and in accordance with our results, it is likely—and more plausible—that empagliflozin exerts an earlier effect compared to the acute effect of dapagliflozin. This discrepancy could be attributed to differences in pharmacokinetics, potency, or selectivity of cardiovascular effects between the two compounds.

Another systematic review and network meta-analysis also suggests that SGLT2i improve endothelial function and arterial stiffness in diabetic individuals. This study highlighted the fact that specific drugs such as dapagliflozin and tofogliflozin showed significant improvements in cf-PWV [20].

These effects might be related to the pleiotropic properties of SGLT2i, which include antioxidant, anti-inflammatory, and vascular remodeling effects [21,23]; it has also been reported that SGLT2i led to a reduction of 73.8% in arrhythmic events among 198 patients in Rome, comparing the events recorded one year prior to and one year following the introduction of SGLT2i therapy for heart failure [24].

In our study, empagliflozin has a significantly greater effect in reducing cf-PWV compared to dapagliflozin, with a highly significant difference (*p* < 0.001). This suggests that the molecule has specific properties that impact arterial stiffness more; these results are consistent with the positive metabolic and hemodynamic effects reported for this molecule; it has been reported in several studies that empagliflozin can improve arterial stiffness parameters [25]. An underlying analysis of a clinical trial showed that empagliflozin reduced central systolic pressure and central pulse pressure, indicating an improvement in arterial stiffness. Another study found that empagliflozin decreased aortic stiffness and improved endothelial function, which could be related to its anti-inflammatory effects [12,17]. These effects on arterial stiffness could contribute to the cardiovascular benefits observed with the use of SGLT2i.

Furthermore, regarding acute variations in cf-PWV levels, we cannot exclude that this different behavior might be due to the different SGLT2 selectivity of the two drugs; These findings are relevant to clinical practice, as they highlight the importance of selecting the appropriate SGLT2 inhibitor treatment to optimize hemodynamic and metabolic outcomes in patients with T2D or related conditions.

### Effect of SGLT2 Inhibitors on Triglyceride Levels

The analysis showed a significant reduction in triglyceride levels in the empagliflozin-treated group compared to the placebo group. This finding is consistent with previous studies that have reported that empagliflozin can significantly reduce triglyceride levels in patients with T2D [26,27]; this improvement in the lipid profile in patients with T2D possibly occurs through indirect mechanisms. These mechanisms include weight loss, improved insulin sensitivity, and mobilization of free fatty acids towards oxidative pathways instead of storage [28]; the results found in our study agree with what has been previously shown.

In contrast, while our study did not find a statistically significant effect of dapagliflozin on triglyceride levels, it is important to acknowledge that other studies and meta-analyses have reported mixed results. For instance, a recent meta-analysis by Bechman et al., 2023 [29] suggests that dapagliflozin may have a modest but significant effect on reducing triglyceride levels in certain patient populations. These discrepancies could be attributed to differences in study design, patient characteristics, treatment duration, or baseline metabolic profiles.

Specifically, the meta-analysis highlights that in some randomized controlled trials, dapagliflozin was associated with a reduction in triglycerides, particularly in patients with higher baseline levels or those with more pronounced insulin resistance. This suggests that the lipid-lowering effects of dapagliflozin may be context-dependent, potentially emerging in subgroups with specific metabolic derangements. Furthermore, the meta-analysis underscores that the magnitude of triglyceride reduction with dapagliflozin, while statistically significant in some studies, tends to be smaller compared to that observed with empagliflozin.

Considering these observations, the lack of a significant effect of dapagliflozin on triglycerides in our study could reflect the specific characteristics of our cohort, such as relatively lower baseline triglyceride levels or shorter treatment duration. Future studies with larger and more diverse patient populations, as well as longer follow-up periods, are needed to clarify the role of dapagliflozin in modulating lipid metabolism and its potential cardiovascular benefits.

These findings imply that intra-class differences among SGLT2 inhibitors may not only be related to their pharmacological properties but also to the metabolic context in which they are used. For example, the degree of obesity, baseline triglyceride levels, and insulin sensitivity could modulate the lipid-lowering effects of dapagliflozin. This variability underscores the importance of personalized approaches when considering the use of SGLT2 inhibitors in clinical practice [30,31].

Although the results are promising, some limitations of the present study should be acknowledged: the observed effects could vary in different subgroups of patients depending on the degree of metabolic control, comorbidities, and baseline characteristics. Duration of the study: the assessment of the change in cf-PWV and triglycerides was performed in a relatively short period. The beneficial effects of empagliflozin on arterial stiffness and its impact on hard cardiovascular events should be confirmed in larger cohort studies. The decrease in triglycerides observed with empagliflozin is clinically relevant, since elevated levels of triglycerides are not statistically significant.

## 5. Conclusions

Short-term treatment with SGLT2 inhibitors acutely reduces arterial stiffness in patients with type 2 diabetes receiving either dapagliflozin or empagliflozin; however, in our study, empagliflozin demonstrated a more significant and faster effect than dapagliflozin, underlining the potential vascular benefits beyond glucose control of SGLT2 inhibitors, particularly related to vascular stiffness, and this may represent an additional benefit in the clinical setting.

## Figures and Tables

**Table 1:** Baseline epidemiological, clinical, and hemodynamic characteristics between intervention groups.

	Intervention Groups			
Baseline Characteristics	Dapagliflozin N = 10 ^1^	Empagliflozin N = 10 ^1^	Placebo N = 10 ^1^	*p* ^2^
Age (years)	51.50 [48.00, 56.00]	47.50 [45.00, 50.00]	51.00 [45.00, 52.00]	0.267
Sex				
Woman	5 (50%)	6 (60%)	7 (70%)	
Man	5 (50%)	4 (40%)	3 (30%)	
Height (cm)	1.62 [1.52, 1.68]	1.64 [1.59, 1.70]	1.58 [1.53, 1.63]	0.452
Weight (kg)	73.80 [72.00, 75.50]	77.45 [73.00, 94.50]	75.85 [73.00, 79.90]	0.527
BMI (kg/m^2^)	27.78 [27.10, 28.42]	29.15 [27.48, 35.57]	28.72 [27.48, 30.07]	0.527
SPB (mmHg)	125.50 [118.00, 134.00]	124.50 [110.00, 134.00]	122.50 [114.00, 128.00]	0.587
DBP (mmHg)	82.50 [76.00, 89.00]	84.00 [75.00, 90.00]	80.50 [73.00, 83.00]	0.490
MAP (mmHg)	95.34 [90.67, 101.33]	97.84 [88.33, 105.33]	93.67 [88.00, 96.67]	0.475
HbA1c (%)	7.95 [7.60, 9.00]	8.95 [8.20, 9.70]	8.85 [8.10, 9.50]	0.101
Glucose (mg/dL)	162.01 [145.00, 180.88]	202.37 [182.00, 304.70]	160.98 [136.30, 180.80]	**0.018**
Triglycerides (mg/dL)	128.05 [116.10, 155.40]	120.05 [115.10, 126.00]	131.50 [113.10, 145.00]	0.754
cf-PWV (m/s)	8.05 [7.10, 8.80]	9.15 [8.50, 9.70]	8.10 [7.15, 9.00]	0.080

^1^ Median [P25, P75]. ^2^ Obtained using the Kruskal–Wallis test. *p* values in bold represent statistical significance (*p* ≤ 0.05). BMI: body mass index. SBP: systolic blood pressure. DBP: diastolic blood pressure. MAP: mean arterial pressure. TG: triglycerides. cf-PWV: carotid femoral pulse wave velocity.

**Table 2:** Epidemiological, clinical, and hemodynamic characteristics between intervention groups after a 7-day treatment intervention.

	Intervention Groups			
Final Characteristics	Dapagliflozin N = 10 ^1^	Empagliflozin N = 10 ^1^	Placebo N = 10 ^1^	*p* ^2^
Age (years)	51.50 [48.00, 56.00]	47.50 [45.00, 50.00]	51.00 [45.00, 52.00]	0.267
Sex				
Woman	5 (50%)	6 (60%)	7 (70%)	
Man	5 (50%)	4 (40%)	3 (30%)	
Height (cm)	1.62 [1.52, 1.68]	1.64 [1.59, 1.70]	1.58 [1.53, 1.63]	0.452
Weight (kg)	72.60 [71.30, 75.10]	72.70 [69.60, 91.00]	76.25 [73.00, 80.60]	0.362
BMI (kg/m^2^)	27.33 [26.84, 28.27]	27.36 [26.20, 34.25]	28.70 [27.48, 30.34]	0.362
SPB (mmHg)	113.93 [109.00, 117.00]	110.50 [101.00, 128.00]	113.50 [111.00, 123.00]	0.741
DBP (mmHg)	73.82 [70.00, 74.63]	72.50 [65.00, 83.00]	78.00 [73.00, 82.00]	0.436
MAP (mmHg)	87.70 [82.33, 87.73]	84.17 [77.00, 98.00]	89.50 [86.67, 92.33]	0.326
Glucose (mg/dL)	130.30 [123.00, 152.00]	155.10 [141.60, 186.00]	161.35 [136.00, 185.00]	0.058
Triglycerides (mg/dL)	133.00 [105.00, 148.20]	105.70 [101.20, 114.00]	135.00 [114.00, 187.00]	**0.033**
cf-PWV (m/s)	7.65 [7.30, 8.50]	8.25 [7.40, 8.40]	8.06 [7.36, 8.50]	0.787

^1^ Median [P25, P75]. ^2^ Obtained using the Kruskal–Wallis U test. *p*-values in bold represent statistical significance (*p* ≤ 0.05) BMI: body mass index. SBP: systolic blood pressure. DBP: diastolic blood pressure. MAP: mean arterial pressure. TG: triglycerides. cf-PWV: carotid femoral pulse wave velocity.

**Table 3:** Cf-PWV change (Δ). Comparison by intervention.

Comparison	Z	*p* Unadjusted	*p* Adjusted	*r*
Dapagliflozin—Empagliflozin	2.909274	0.004	0.005 ^†^	0.650
Dapagliflozin—Placebo	−1.804004	0.071	0.071	0.403
Empagliflozin—Placebo	−4.713278	<0.001	<0.001 ^¥^	1.054

Obtained using a Dunn post hoc analysis. *p*-values: <0.05 ^†^, <0.001 ^¥^.

## Data Availability

The original contributions presented in this study are included in the article. Further inquiries can be directed to the corresponding author.

## Abbreviations

The following abbreviations are used in this manuscript:

ACEI	Angiotensin-Converting Enzyme Inhibitors
ARAII	Angiotensin II Receptor Antagonists
BMI	Body Mass Index
BP	Blood Pressure
cf-PWV	Carotid–Femoral Pulse Wave Velocity
DBP	Diastolic Blood Pressure
ECG	Electrocardiogram
GFR	Glomerular Filtration Rate
HbA1c	Glycated Hemoglobin
SGLT2i	Sodium-Glucose Cotransporter-2 Inhibitors
MAP	Mean Arterial Pressure
PWV	Pulse Wave Velocity
SBP	Systolic Blood Pressure
SGLT2	Sodium-Glucose Cotransporter-2
T1D	Type 1 Diabetes
T2D	Type 2 Diabetes
TG	Triglycerides
TZD	Thiazolidinediones
UTI	Urinary Tract Infection
